# A Complex Case of Pituitary Apoplexy Mimicking a Stroke: A Case Report With a Review of the Literature

**DOI:** 10.7759/cureus.65332

**Published:** 2024-07-25

**Authors:** Lyubomir Gaydarski, Deyan Popov, Danny Kolev, Asen Hadzhiyanev

**Affiliations:** 1 Department of Anatomy, Histology and Embryology, Medical University of Sofia, Sofia, BGR; 2 Clinic of Neurosurgery, University Multiprofile Hospital for Active Treatment (UMHAT) ‘’St. Ivan Rilski’’, Sofia, BGR; 3 Department of Neurosurgery, Medical University of Sofia, Sofia, BGR

**Keywords:** treatment, imaging, diagnostics, pituitary macroadenoma, pituitary apoplexy

## Abstract

Pituitary adenomas, benign tumors originating in the pituitary gland, are classified based on size and hormone activity. Pituitary apoplexy, defined by hemorrhage or infarction of these adenomas, poses a challenge in estimation due to its subtle presentation. It often manifests as sudden, severe headaches, visual disturbances, nausea, and hormone deficiencies. Diagnosis typically involves MRI or, if unavailable, CT scans, alongside hormonal and other laboratory assessments. Here, we present the case of a 71-year-old female patient who was transferred to the hospital for endovascular treatment of a hemorrhagic stroke after presenting with sudden-onset symptoms suggestive of a stroke. Neurological examination revealed right-sided ptosis, diplopia, and bitemporal hemianopsia. The initially performed CT imaging suggested hemorrhagic stroke. Subsequent MRI reviewed pituitary macroadenoma with apoplexy. Immediate surgical management via endoscopic, endonasal, transsphenoidal adenomectomy resulted in gradual symptom improvement. The patient was discharged three days after the procedure with complete symptom improvement and showed no signs of recurrence during regular follow-ups at one and three months post-surgery. This case emphasizes the critical role of accurate imaging in diagnosing and managing patients with pituitary macroadenomas complicated by apoplexy, which can mimic stroke. While CT scans may lack detail, MRI offers precise information. Prompt recognition and evaluation are vital for effective treatment, highlighting the importance of distinguishing between these conditions for optimal patient care.

## Introduction

Pituitary adenomas are benign tumors originating in the pituitary gland. Pituitary adenomas can be categorized by size. Microadenomas are tumors smaller than 10 mm, while tumors larger than 11 mm are called macroadenomas [1]. Recent research has enhanced our understanding of the various hormonal profiles exhibited by these tumors, which can be further categorized as hormone-secreting and non-secreting. In the last classification of the World Health Organization, pituitary adenomas are depicted in greater detail based on their immunoreactivity [1]. Pituitary adenomas are slow progressive tumors, and recent research by Hadzhiyanev et al. demonstrated a correlation between the size of the pituitary adenoma and MIB-1 activity, on the one hand, and the expression of p53 and the invasiveness and progression of the adenoma, on the other hand [2].

Pituitary apoplexy is an uncommon medical emergency defined by hemorrhage or infarction of a preexisting pituitary adenoma [3]. Estimating its prevalence and incidence is challenging due to the subtle presentation of many subacute cases, which often remain undiagnosed. Nonetheless, it is estimated to have a prevalence of 62 cases per million people [4] and an incidence of 1.04 cases per million per year [5]. Research indicates that pituitary apoplexy occurs in approximately 2-12% of patients with pituitary adenomas [3]. In 80% of symptomatic cases, apoplexy serves as the initial manifestation of a previously undiagnosed pituitary adenoma [6]. The hallmark symptoms of pituitary apoplexy include headache, visual disturbances, nausea, and pituitary hormone deficiencies [3,6]. Clinically, pituitary apoplexy presents with acute symptoms such as a sudden, severe headache, nausea, vomiting, visual disturbances, and ophthalmoplegia caused by the involvement of cranial nerves III, IV, and VI. In severe cases, there may be a decline in the level of consciousness [7]. Imaging studies are necessary to diagnose pituitary apoplexy. MRI is the preferred method, with a sensitivity of approximately 90% [8]. If MRI is contraindicated or unavailable, CT can be the only diagnostic method. CT scans are effective at visualizing expansile pituitary lesions that cause enlargement of the sella turcica, as well as acute parenchymal hemorrhages and hemorrhagical strokes. While CT detects proliferating pituitary lesions in over 80% of cases, it only diagnoses fewer than 30% of pituitary apoplexy cases [9]. Additionally, pituitary hormone levels, electrolytes, renal and liver function, coagulation, blood count, and specific hormone levels should be urgently measured to detect any deficiencies or endocrine conditions related to hormone hypersecretion [10]. Finally, it is crucial to distinguish pituitary apoplexy from other conditions that can present with similar symptoms, such as internal carotid artery aneurysmal rupture (mainly of the cavernous segment of the internal carotid artery), bacterial meningitis, subarachnoid hemorrhage, and adult optic neuritis [11,12]. The optimal treatment for pituitary apoplexy is debated, with some studies advocating for early surgical intervention and others for a conservative approach depending on symptom severity. Symptoms can vary widely, necessitating individualized treatment plans coordinated by a multidisciplinary team. Promptly diagnosed patients with larger adenomas and neurological deficits are indicated for immediate surgical treatment [12]. Pituitary apoplexy is a rare and not fully understood emergency condition. This case report aims to present a complex instance of pituitary apoplexy that mimicked a hemorrhagic stroke and to share our experience in diagnosing and managing such a case.

## Case presentation

А 71-year-old female patient was transferred to our hospital for diagnostic evaluation. The patient presented with sudden-onset symptoms suggestive of a stroke. She experienced severe right frontal headache, nausea, and vomiting, followed by a dropping of the right eyelid. The patient reported a history of type 2 diabetes and arterial hypertension. She denied any family history of diabetes or hypertension, and she also stated that she had no allergies. Upon admission to the emergency department, an initial diagnosis of stroke was considered. Neurological examination revealed right-sided ptosis, diplopia, bitemporal hemianopsia, and right eye deviation upwards and outwards, suggesting possible paralysis of the oculomotor and trochlear nerves. Pupillary reflexes were intact, and no other pathological reflexes were detected. CT was performed in the local emergency department before transferring her to the Specialized Hospital - Neurosurgery Center and Center for the Treatment of Cerebrovascular Diseases. The CT revealed a heterodense intrasellar formation. Blood samples were taken from the patient, and the results are summarized in Table 1.

**Table 1:** Results of the lab test conducted upon admission.

Parameters (units)	Result	Reference range
Alanine transaminase (U/L)	17	0–50
Aspartate transaminase (U/L)	23	0–50
Gamma-glutamyltransferase (U/L)	34	0–30
Glucose (mmol/L)	14.8	4.1–5.9
Potassium (mmol/L)	3.8	3.5–5.1
Sodium (mmol/L)	133	136–146
Urea (mmol/L)	4.6	2.8–7.2
Chloride (mmol/L)	100	98–111
Total protein (g/L)	63	60–83
Creatinine (µmol/L)	64	74–110
Estimated glomerular filtration rate (mL/minute)	88	60–120
Prothrombin time (seconds)	12.9	8.0–13.2
Prothrombin time (%)	97.5	80–120
International normalized ratio	1.1	0.8–1.2
Activated partial thromboplastin time (seconds)	23.1	30–40
Fibrinogen	3.0	1.5–4.5
White blood cell (10^9^/L)	6.22	4.1–11.0
Lymphocytes (10^9^/L)	1.53	0.6–4.1
Lymphocytes (%)	24.7	20–40
MID (10^9^/L)	1.1	0.0–1.8
MID% (%)	5.5	0–10
Granulocyte (10^9^/L)	6.4	2.0–7.8
Granulocyte (%)	69	54–76
Red blood cell (10^12^/L)	3.88	4.6–6.3
Hemoglobin (g/L)	116	120–160
Hematocrit (L/L)	0.35	0.40–0.51
Mean cell volume (fL)	91	82–98
Mean cell hemoglobin (pg)	29.9	26.0–32.0
Mean cell hemoglobin concentration (g/L)	330	300–360
Red cell distribution width (%)	13.6	11.5–14.5
Platelet (10^9^/L)	320	140–440

**Figure 1:**
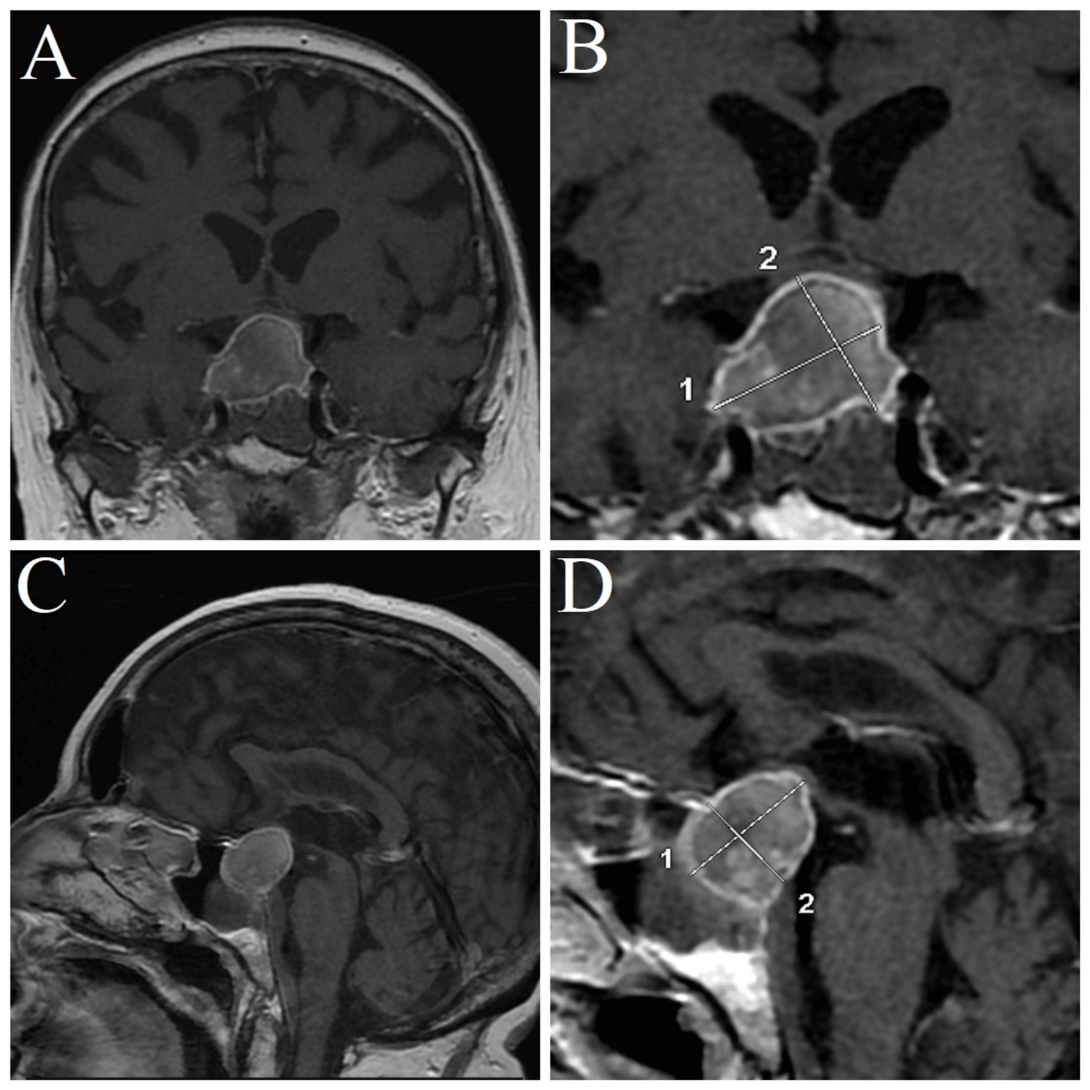
MRI results. (A) MRI in the coronal plane demonstrating pituitary macroadenoma with apoplexy and compression of the optic chiasm and the cavernous sinus bilaterally. (B) Zoomed in on the adenoma. 1 = 29.2 mm, 2 = 24.5 mm. (C) MRI in the sagittal plane demonstrating pituitary macroadenoma with apoplexy, with compression of the optic chiasm. (D) Zoomed in on the pituitary adenoma. 1 = 27.6 mm, 2 = 20.1 mm.

Consultation with a neurosurgeon and subsequent MRI reviewed a hyperintense intrasellar formation, which measured 27.6 × 20.1 mm in the sagittal plane and 29.2 × 24.5 mm in the coronal plane. The formation had both hyperintense and hypointense zones, which aligned with the radiological description of pituitary adenoma with apoplexy. Moreover, the register formation was causing compression of the optic chiasm and the adjacent right cavernous sinus (Figure 1).

**Figure 2:**
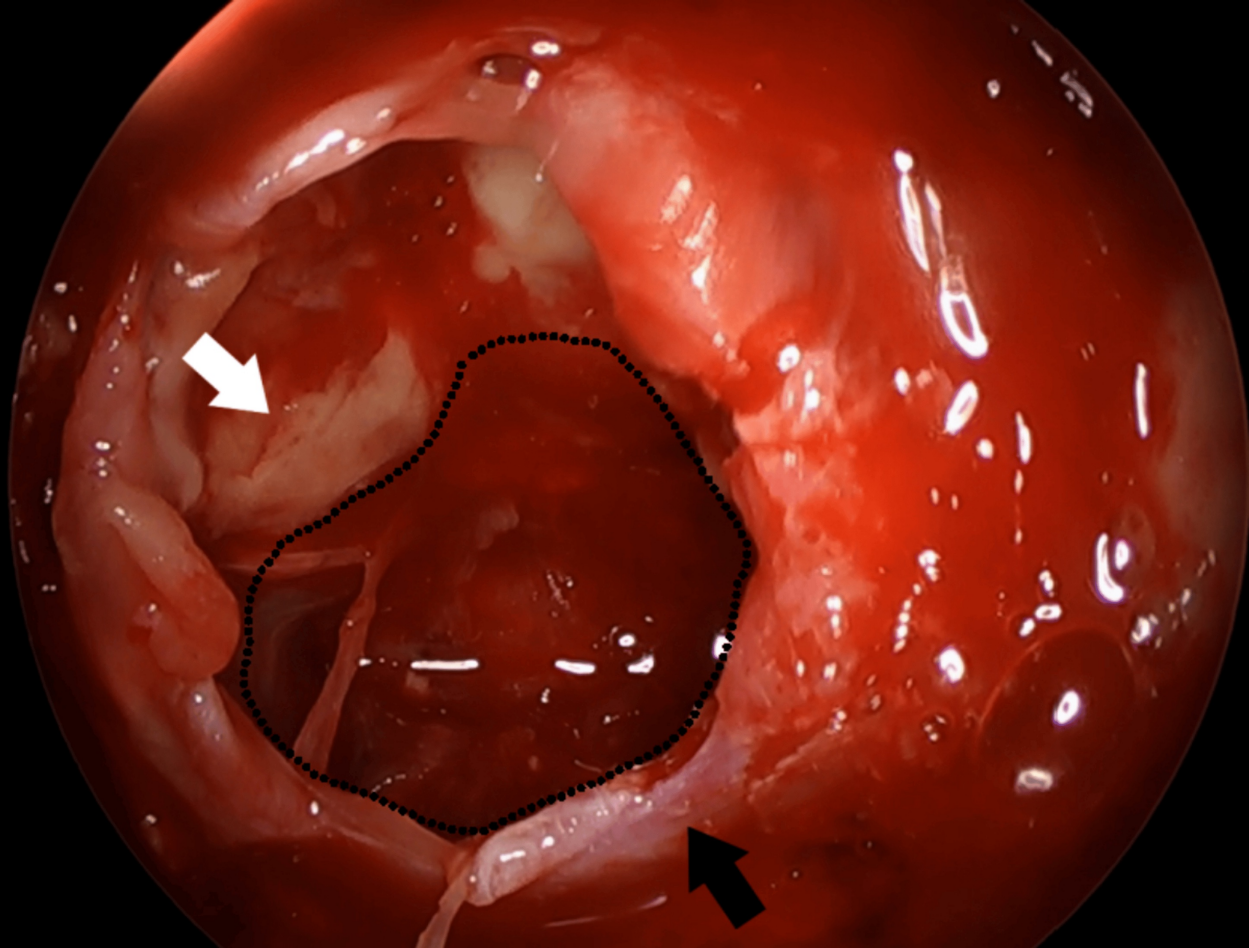
Intraoperative view of the opened sella turcica filled with blood and peripherally situated parts of the pituitary adenoma. Black arrow: the base of sella turcica. White arrow: pituitary gland. Black dotted line: marking the borders of the space infilled with dark stained blood before opening the dura.

The patient underwent immediate surgical management with an endoscopic, endonasal, transsphenoidal adenomectomy. Upon opening the anterior wall of the sella turcica and gently opening the dura, we observed the evacuation of dark-colored blood collected within the sella (Figure 2).

Subsequently, a complete adenectomy was performed, followed by meticulous endoscopic inspection and hemostasis. No intraoperative complications were encountered. After the surgery, the symptoms gradually improved, with both ptosis and diplopia resolved by the following day. No endocrine deficiencies were present. The results of the postoperative blood tests are summarized in Table 2.

**Table 2:** Results of the postoperative blood samples.

Parameters (units)	Result	Reference range
Alanine transaminase (U/L)	13	0–50
Aspartate transaminase (U/L)	19	0–50
Gamma-glutamyltransferase (U/L)	28	0–30
Glucose (mmol/L)	6.3	4.1–5.9
Potassium (mmol/L)	3.5	3.5–5.1
Sodium (mmol/L)	140	136–146
Urea (mmol/L)	2.4	2.8–7.2
Chloride (mmol/L)	108	98–111
Total protein (g/L)	61	60–83
Creatinine (µmol/L)	58	74–110
Estimated glomerular filtration rate (mL/minute)	91	60–120
Prothrombin time (seconds)	13.1	8.0–13.2
Prothrombin time (%)	99.8	80–120
International normalized ratio	1.2	0.8–1.2
Activated partial thromboplastin time (seconds)	24.6	30–40
Fibrinogen	3.9	1.5–4.5
White blood cell (10^9^/L)	4.69	4.1–11.0
Lymphocytes (10^9^/L)	1.86	0.6–4.1
Lymphocytes (%)	29.7	20–40
MID (10^9^/L)	1.3	0.0–1.8
MID (%)	6.3	0–10
Granulocyte (10^9^/L)	6.6	2.0–7.8
Granulocyte (%)	70	54–76
Red blood cell (10^12^/L)	3.53	4.6–6.3
Hemoglobin (g/L)	105	120–160
Hematocrit (L/L)	0.32	0.40–0.51
Mean cell volume (fL)	92	82–98
Mean cell hemoglobin (pg)	29.6	26.0–32.0
Mean cell hemoglobin concentration (g/L)	323	300–360
Red cell distribution width (%)	13.5	11.5–14.5
Platelet (10^9^/L)	323	140–440

The patient was discharged three days after the procedure with complete symptom improvement. Regular follow-ups at one and three months post-surgery showed no signs of recurrence.

## Discussion

The present article features a complex case of pituitary apoplexy presenting as a stroke and highlights the difficulties of correctly diagnosing and managing such an emergency patient with acute neurological deficit. The presented case was especially challenging for diagnostics in a smaller center without MRI and neurosurgery. Based on the results of the MRI, the presented adenoma was classified as a macroadenoma and could be further classified as type 3b by the Knosp classification as it extended laterally to the internal carotid artery and into the inferior compartment of the cavernous sinus [13]. The observed oculomotor deficiency was due to cavernous compression. In certain instances, the extent of necrosis from an apoplectic event can be so significant that it eradicates the hormone-producing tumor cells, which might hide a previously hormone-secreting adenoma [14]. However, the patient in this case exhibited no clinical signs indicative of a functioning adenoma. Overall, pituitary apoplexy with neurological symptoms is rarely encountered, yet it is a potentially life-threatening complication of preexisting pituitary adenoma [14]. Puglisi et al. reported a case of a giant pituitary adenoma with apoplexy, which presented altered mental status and bitemporal hemianopsia. In their case, the adenoma measured nearly 6 cm in diameter (twice bigger than ours), and the patient was 81 years old, 10 years older than our case. The patient was managed conservatively due to the age and condition, and the authors reported that hormone-replacing therapy significantly improved the neurological status and endocrinological manifestations [14]. Pokhrel et al. recently reported a rare case of pituitary apoplexy complicated with cerebral infarction. In their case, a 28-year-old patient presented with stroke-like symptoms, including right-sided hemiparesis, facial palsy, and ptosis. The authors theorized that the observed cerebral ischemia was a result of the compression syndrome caused by the pituitary apoplexy [15]. Even though the authors did not specify the dimension of the adenoma, based on the provided CT and MRI, we can classify the adenoma as a giant adenoma type 4 based on Knosp classification. This case shows that such a diagnosis is possible even in young patients. Moreover, Boscain and Montagnese recently reported an intriguing case of pituitary apoplexy presenting as myocardial infarction and stroke. The adenoma measured 15 × 16 × 20 mm, and the authors hypothesized that the observed syndromes resulted from the endocrine and, more specifically, adrenal deficit due to the PA. The 80-year-old patient was managed conservatively due to age and related comorbidities, with promising results [16]. However, the reported adenoma was considerably smaller than the one we present.

Pituitary apoplexy often occurs in patients with no prior history of a pituitary mass, making timely diagnosis difficult due to its resemblance to various neurological disorders and other critical conditions, frequently resulting in delayed identification and treatment [14]. The primary differential diagnoses to consider are subarachnoid hemorrhage (SAH) from aneurysm rupture and bacterial meningitis. Additional conditions to rule out include suprasellar aneurysm, ruptured aneurysm leading to SAH, intracerebral hemorrhage, hypertensive encephalopathy, and cavernous sinus thrombosis [17]. Symptoms of mechanical compression and endocrine dysfunction typically develop within a few hours to 48 hours following the onset of apoplexy, although a subacute progression is also possible [3]. MRI/MRA is the preferred method for diagnostic cranial imaging. During the initial 24 hours, T2-weighted images show hypointense changes due to deoxyhemoglobin. In subacute and chronic stages of pituitary apoplexy, T1-weighted images display focal hyperintensity, while T2-weighted images reveal reduced and increased signal intensity areas. A fluid level may become visible within two weeks [8].

Pituitary apoplexy is a complex pathology requiring rapid management, typically surgically or, in selected cases, conservatively, necessitating the involvement of a multidisciplinary team for decision-making. If the correct treatment is delayed because of late diagnosis (due to lack of MRI and/or neurosurgical consultation or different reasons), it will result in worsened recovery chances, even potential disability or death [18]. Surgical treatment of pituitary apoplexy typically involves both microscopic or endoscopic transsphenoidal approaches [18,19]. Recent research by Popov et al. [18] and Hadzhiyqnev and Popov [19] conducted in our country, and based on our institutional experience, suggests that the endoscopic approach provides better visualization and excision opportunity of the adenoma with lower possibility of intraoperative complications [18,19]. Furthermore, the endoscopic approach has been proven to lead to statistically significantly fewer recurrences of invasive pituitary adenomas [18], as presented in our current case. However, due to the tumor’s extension and involvement of neurovascular structures, surgery often poses significant challenges [20]. Nevertheless, decompression and total adenomectomy are preferred in patients who were promptly diagnosed with pituitary apoplexy [12]. Similarly, in our case, we observed rapid improvement in the patient’s symptoms due to the immediate surgical management.

## Conclusions

The present case underscores the importance of comprehensive imaging diagnostics for patients presenting with sudden neurological deficits. While CT scans may lack detailed information and lead to misinterpretations, MRI provides more accurate insights. The case of a 71-year-old female patient, who exhibited stroke-like symptoms and was successfully treated with endoscopic, endonasal, transsphenoidal adenomectomy, highlights this point. This approach led to complete symptom resolution and no recurrence at follow-ups. Our case highlights the necessity for prompt recognition and precise evaluation, which are crucial for the effective management of patients with pituitary macroadenomas complicated by apoplexy, a condition that can clinically mimic stroke. Treatment must be conducted in a specialized center equipped with the necessary technical equipment and trained personnel.
